# Characterization of the first two toxins isolated from the venom of the ancient scorpion *Tityus (Archaeotityus) mattogrossensis* (Borelli, 1901)

**DOI:** 10.1590/1678-9199-JVATITD-2021-0035

**Published:** 2021-12-13

**Authors:** Natiela Beatriz de Oliveira, Ana Carolina Martins Magalhães, Carlos Bloch, Paulo Sérgio Lacerda Beirão, Anita de Oliveira Silva, Rafael D. Melani, Eder Alves Barbosa, Osmindo Rodrigues Pires, Carlos Alberto Schwartz

**Affiliations:** 1 Laboratory of Toxinology, Department of Physiological Sciences, Institute of Biology, University of Brasilia (UnB), Brasília, DF, Brazil.; 2 EMPRABA Center of Genetic Resources and Biotechnology, Brazilian Agricultural Research Corporation, Brasília, DF, Brazil.; 3 Department of Biochemistry and Immunology, Institute of Exact Sciences, Federal University of Minas Gerais (UFMG), Belo Horizonte, MG, Brazil.; 4 Laboratory of Biomolecule Synthesis and Analy sis, Institute of Chemistry, University of Brasilia (UnB), Brasília, DF, Brazil.

**Keywords:** Archaeotityus, Scorpion, Tityus (Archaeotityus) mattogrossensis, Toxins

## Abstract

**Background:**

Almost all *Tityus* characterized toxins are from subgenera *Atreus* and *Tityus*, there are only a few data about toxins produced by *Archaeotityus*, an ancient group in *Tityus* genus.

**Methods:**

*Tityus (Archaeotityus) mattogrossensis* crude venom was fractionated by high performance liquid chromatography, the major fractions were tested in a frog sciatic nerve single sucrose-gap technique. Two fractions (Tm1 and Tm2) were isolated, partially sequenced by MALDI-TOF/MS and electrophysiological assayed on HEK293 Nav 1.3, HEK293 Nav 1.6, DUM and DRG cells.

**Results:**

The sucrose-gap technique showed neurotoxicity in four fractions. One fraction caused a delay of action potential repolarization and other three caused a reduction in amplitude. An electrophysiological assay showed that Tm1 is active on HEK293 Nav 1.3, HEK293 Nav 1.6, DUM and DRG cells, and Tm2 on HEK293 Nav 1.3 and DRG cells, but not in HEK293 Nav 1.6. In addition, Tm1 and Tm2 did promote a shift to more negative potentials strongly suggesting that both are α-NaScTx.

**Conclusion:**

Although *Tityus (Archaeotityus) mattogrossensis* is considered an ancient group in *Tityus* genus, the primary structure of Tm1 and Tm2 is more related to *Tityus* subgenus. The patch clamp electrophysiological tests suggest that Tm1 and Tm2 are NaScTx, and also promoted no shift to more negative potentials, strongly suggesting that both are α-NaScTx. This paper aimed to explore and characterize for the first time toxins from the ancient scorpion *Tityus (Archaeotityus) mattogrossensis.*

## Background

All scorpion species considered dangerous to man belong to the Buthidae family, the only family geographically distributed throughout all continents. It is estimated that there are approximately 1228 species in Buthidae belonging to approximately 80 genera, six of them with medical importance: *Androctonus*, *Centruroides*, *Hottentotta*, *Leiurus*, *Parabuthus* and *Tityus* [1]*. Tityus* is a neotropical scorpion genus and the most diverse in Buthidae family with more than 200 described species [2], being especially diversified in Central and South America tropical areas, only a few species occur in temperate South America [3]. 

Due the intrageneric morphological variation, *Tityus* genus is the most taxonomically confusing in Buthidae, making a taxonomic revision necessary [4]. Based on morphological characters Lourenço [5] suggested to separate *Tityus* into five subgenera:

- *Archaeotityus* proposed to gather the species currently reported to the group *'Tityus clathratus'* species and scorpions from columbianus group of Kraepelin (1911), formed by small and irregularly painted forms;
- *Atreus* that covers species from *'Tityus asthenes'* group and Cambridgei group of Kraepelin (1911), composed of large, reddish-brown to black in color specimens;
- *Brazilotityus* that can be distinguished from *Tityus* by the following characters: (i) combs much smaller than those found in *Tityus* species, with particularly reduced fulcers, (ii) sharp edges of the fixed and mobile fingers of the hands of the pedipalps with 10-12 / 11-13 sets of granules;
- *Caribetityus* created to gather some species of *Tityus* of Grandes Antilles, distributed in particular in the Dominican Republic and it has as its type species *Caribe Tityus elii;*
- *Tityus* that comprises species like *Tityus bahiensis*.

*Archaeotityus* includes 24 valid species distributed in the Neotropical region from Costa Rica to central Argentina. The *Archaeotityus* species share a small size (20-40 mm), general coloring reticulated on a background ranging from pale yellow to reddish and a medium to large pyramidal subaculular tubercle [6]. According to Lourenço [5], *Archaeotityus* constitutes the ancient group occupying a plesiomorphic position, because the variegated pigmentation and median dorsal carina strongest distal tooth are considered primitive characters and found only in the juvenile stages of *Tityus* species. The *Tityus* characterized toxins are almost totally from subgenera *Atreus* and *Tityus* species, there is scarce data about toxins produced by *Archaeotityus* [7]*.*

The endemic Brazilian Cerrado scorpion *Tityus (Archaeotityus) mattogrossensis* (Borelli, 1901) is found in the Brazil Midwest, Bahia and north of Minas Gerais states [8]*. Tityus (Archaeotityus) mattogrossensis* sting is considered of small severity due to the low degree of reaction and the few recorded cases [9]. The accident with species of the subgenus *Archaeotityus* is considered mild and moderate, commonly presenting pain at the sting site shortly after the accident and paresthesia in the affected area. In some cases, the victims also experienced chills, dizziness, headache and vomiting [9,10].

The present study aims to describe the first two *Tityus (Archaeotityus) mattogrossensis* toxins and their electrophysiological activity in Nav 1.3, Nav 1.6, DUM and DRG cells, enhancing knowledge on relationship of *Archaeotityus* with other *Tityus* NaScTx.

## Methods

**Figure1.**
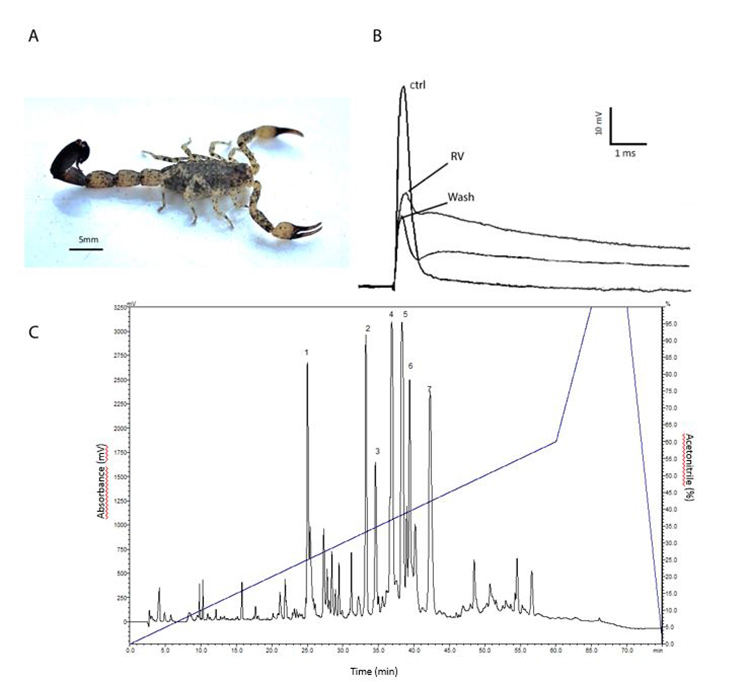
(A) Specimen of *Tityus (Archaeotityus) mattogrossensis.* Photo by Osmindo R. P. Júnior. (B) Effect of *Tityus (Archaeotityus) mattogrossensis* crude venom (0.754 mg/mL) on the action potential of the frog sciatic nerve, using the single sucrose-gap technique. Ctrl: control; RV: potential reduction after 1 minute of application of *Tityus (Archaeotityus) mattogrossensis* crude venom; Wash: after 10 minutes of washing, showing that the action is irreversible (n = 1). (C) Chromatogram of *Tityus (Archaeotityus) mattogrossensis* crude venom (10 mg/mL). The seven most abundant chromatographic fractions used in this work are indicated. C18 Phenomenex Synergi Hydro analytical column (4.60 x 250 mm) and the fractions detected at 216 nm, flow of 1 mL/min and acetonitrile gradient according to the methodology described in the text.

The specimens (n = 60) of *Tityus (Archaeotityus) mattogrossensis* (Figure 1A) were collected in the municipality of Jaborandi, Bahia, Brazil (13º37'10" S 44º25'58" W) (collection permission - SISBIO: 23408-1 and SISGEN A826A3A), with the help of LED-UV lanterns (395 nm). After collection, the animals were kept in containment boxes in vivariums of the Institute of Biology at University of Brasilia, Brazil, with adequate humidity and temperature, with water "ad libitum" and periodically fed with cockroaches.

The venom extraction was performed through an electrical stimulus (from 50 to 100 V, 300 Hz) close to the telson. The venom was collected in polyethylene tubes, solubilized in 1 mL of deionized water and centrifuged for 10 min at 10,500 xg, at room temperature. The quantification of the crude venom was performed using a spectrophotometer (absorbance 280 nm), assuming that an absorbance unit in a 1 cm quartz cuvette is equivalent to 1 mg/mL of protein concentration [11]. Then, the crude venom was vacuum dried (SPD2020 Integrated SpeedVac Concentrator, Thermo Fisher Scientific) and kept refrigerated (-20 °C) for further analysis.

### Venom purification - RP-HPLC

Crude venom aliquots (10 mg/mL) containing 0.12% trifluoroacetic acid (TFA) were applied in a reverse phase high performance liquid chromatography system (RP-HPLC): Shimadzu Co. (Kyoto, Japan) LC10A series; equipped with SPD-M10A diode array and C18 Phenomenex Synergi Hydro-RP analytical column (4.60 x 250 mm, 4 µm) with binary solvent gradient: solvent (A) 0.12% TFA aqueous solution and solvent (B) acetonitrile solution and 0.1% TFA (v/v), with a variation from 0 to 60% of B in 60 min; from 60 to 100% B in 5 min 100% B in 5 min, 100 to 0% B in 5 min with absorbance monitoring at 216 and 280 nm and with a flow rate of 1 mL/min. The fractions were manually collected, vacuum dried (SPD2020 Integrated SpeedVac Concentrator, Thermo Fisher Scientific) and stored at -20 °C.

For further analysis, F5 (Tm1) and F6 (Tm2) were submitted to other chromatographic step on RP-HPLC system using analytical column Phenomenex Luna C18 (4.60 x 150 mm, 3 μm), optimized gradient of acetonitrile was used: 0-25% of B in 5 min, followed by 25-35% of B for 20 minutes, 35-100% of B for 5 minutes and 100% of B for more 5 minutes; monitored at 216 and 280 nm. 

The fractions were manually collected and quantified by calculating the detection at 216, 280 and 340 nm from the NanoVue spectrophotometer (GE Healthcare, Sweden) using the protein methodology provided by the manufacturer and kept dry at -20ºC for later analysis.

### Mass spectrometry

MALDI-TOF/MS analyses were performed on MALDI-TOF system mass spectrometers (ULTRAFLEX III and AUTOFLEX SPEED, Bruker Daltonics, Billerica, MA, USA) in positive linear and reflected modes. The 7 more abundant fractions (F1 to 7) from the crude venom HPLC purification were applied to a MALDI-TOF analysis plate followed by the addition of saturated solution of α-cyano-4-hydroxycinnamic acid matrix (HCCA), the following calibrators were used: Peptide calibration standard II for low mass and Protein calibration I for high mass both from the Bruker Daltonics Inc. Nitrogen laser was used at an intensity of 20 to 40%. 

The monoisotopic masses of high molecular weight peptides were elucidated with the use of the ionization mass spectrometer by Electrospray coupled to a quadrupole-TOF system (MicrOTOF-QII, Bruker Daltonics, Germany), operated in positive mode. The samples were dissolved in a solvent with 50% acetonitrile, 47.3% deionized water and 2.6% formic acid and injected into the system by a 180 µL/min continuous flow pump.

### *De novo* sequencing and phylogenetic tree construction

For the *de novo* sequencing F5 (Tm1) and F6 (Tm2) were analysed with and without trypsin digestion: spectra of intact proteins were acquired by using the In Source Decay (ISD) method using the reducing matrix 1,5-diaminonaphthalene (DAN), which mainly results on *c-* and *z-* ions [12], bovine serum albumin (BSA) as calibrator was used.

The second technique used trypsin digestion: the peptides were reduced by dithiothreitol-DTT (25 mM DTT in 100 mM NH_4_HCO_3_) for 1 h in continuous agitation at 60ºC and alkylated by Iodoacetamide (25 mM Iodoacetamide also diluted in solution of 100 mM NH_4_HCO_3_), for 40 min with continuous agitation at 37ºC. The samples were digested in 50 mM immobilized Trypsin (TPKC Treated- Thermo Fisher Scientific) in 100 mM NH_4_HCO_3_ buffer, incubated at 37ºC from 2 to 4h [12].

The tryptic fragments were analysed in MALDI-TOF/MS and fragmented using LIFT mode. The sequences were interpreted using Flex Analysis 3.3 software. 

BLAST suite (http://blast.ncbi.nlm.nih.gov/Blast.cgi?PAGE=Proteins) was used for sequence similarity search in NCBI protein database Clustal Omega (https://www.ebi.ac.uk/Tools/msa/clustalo/) was used to alignment the mature toxins sequences obtained with Tm1 and Tm2.

The Phylogeny tree was performed by Simple Phylogeny tool (https://www.ebi.ac.uk/Tools/phylogeny/simple_phylogeny/) after the sequences alignment. Conditions: Tree Format Default, Distance Correction Off, Exclude Gaps Off, Clustering Method Neighbour-Joining and P.I.M. Off.

### Electrophysiology

*Single sucrose gap*

The activity of *Tityus (Archaeotityus) mattogrossensis* crude venom (0.754 mg/mL) or RP-HPLC fractions (0.4 µg/mL of protein) were tested on the action potential of the frog sciatic nerve (*Lithobates catesbeianus*), an adaptation of the “single sucrose-gap” technique described in [13]. This work was approved by the Animal Use Ethics Committee of University of Brasilia, number 124645/2011.

The frog was euthanized, the sciatic nerve was removed and the connective tissue surrounding it was carefully removed. The nerve was placed through 5 cell chambers, mechanically and electrically isolated with vaseline seals. The first two chambers were used for supramaximal stimulation, pulses from 6 to 7 V, 25 ms, generated by the stimulator S8 - Grass Instruments. In the third, was the assay chamber, which 350 µL of the venom fraction were applied, after the assay this chamber also can be constantly washed by an infusion system. In the fourth chamber a 216 mM sucrose solution was placed and constantly renewed with an infusion system. With the exception of chamber 4, all other chambers were filled with saline solution for amphibians - Ringer (111 mM NaCl, 1.9 mM KCl, 2.4 mM NaHCO_3_ and 1.1 mM CaCl_2_) keeping the nerve submerged. Action potentials were captured in chambers 3 and 5 by Ag-Cl electrodes connected to a high impedance DC differential amplifier with a 50-fold gain coupled to a Tektronix TDS 360 digital oscilloscope (Tektronix, USA). Records were made every 5 min after the sample application, for a total of 40 min, and every 5 minutes after the start of the washing of the assay chamber, for a total of 30 min. These records were compared with the control record obtained before applying the sample. The tests took place at room temperature and the samples to be were resuspended 350 µL of Ringer's solution [14]. 

*Whole cell patch clamp*

The micropipettes were made from glass capillaries using a drawing board (model PP-830, Narishige, Japan) and were filled with the internal solution according to each cell type. The records of ionic currents were made with 1-3 MΏ resistance, in an EPC 9- Patch Clamp Amplifier (HEKA, Germany). Current responses were recorded using the PatchMaster, HEKA software and analyzed with Sigma Plot 10.0 software.

The Tm1 and Tm2 peptides isolated from the *T. (Archaeotityus) mattogrossensis* venom were dissolved in a specific external solution for each tested cell (1 µM of each peptide) and microperfused throughout the experiment using a micropipette placed close to the recorded cell. The experiments were carried out at room temperature (25 ± 2 °C).

The Patch Clamp was recorded in four different cell types: immortalized HEK293 cells (human embryo kidney) that express only Nav 1.3 channels and HEK293 cells that express only Nav 1.6; DRG cells (rat Dorsal Root Ganglion); and DUM cells (Dorsal Unpaired Median from cockroach).

*HEK293 cells*

HEK cells were cultured in DMEM medium with 4.5 g/L glucose, 10% fetal bovine serum and 1% antibiotic (5000 units of penicillin and 5 mg of streptomycin/mL) and incubated at 37 ºC in an atmosphere of 5% CO_2_. The cells were bathed with the external solution (140 mM NaCl, 2 mM CaCl_2_, 1 mM MgCl_2_, 15mM Glucose and 10 mM HEPES, pH 7.4) and the micropipette filled with the internal solution (130 mM CsCl, 1 mM MgCl_2_, 10 mM HEPES,10 mM EGTA, 5 mM NaCl, pH 7.2).

Two electrophysiological protocols were used, the first, called "test 0", the cell was maintained at the holding potential of -80 mV, then a pulse of -120 mV with a duration of 100 ms was applied, so that all sodium channels were in the closed state, and just after a test pulse of 0 mV for 50 ms. The second, a -120 mV prepulse was applied with a duration of 100 ms and starting from the potential of -80 mV in increments of 10 mV (up to +80 mV) for 50 ms, to obtain a current/voltage ratio.

To complement the analysis of Tm1 and Tm2 activity on the Nav channels, the same "0 test" and current vs voltage protocols were performed, but with a depolarizing pulse of +50 mV for 1 ms before applying the 0mV pulse as in the previous protocol.

*DRG cells*

To isolate rat dorsal root ganglion cells (DRG), a male Wistar rat (approximately 250 g) was beheaded by guillotining and all dorsal root ganglia were removed and placed in 0.9% NaCl solution. Subsequently, they went through two enzymatic processes for cell dissociation: in the first, the ganglia were placed for 20 min at 37 ºC in a solution of 3 mg papain and 2 crystals of cysteine ​​diluted in 3 mL of mammalian ringer without Ca^2+^ and Mg^2+^ (140 mM NaCl, 2.5 mM KCl, 10 mM HEPES, 7.5 mM glucose, pH 7.4), subsequently centrifuged at 800 xg for 1 min and the supernatant discarded. The same procedure was carried out in the second enzymatic process, however with 7.5 mg type II collagenase enzyme from *Clostridium histolyticum* diluted in 3 mL of ringer. The cells were washed and mechanically crushed in HAM F-12 culture medium, 1% antibiotic (5000 units of penicillin and 5 mg streptomycin/mL), plated and, after 2 h, fed with culture medium L-15, 0.5% antibiotic (5000 units of penicillin and 5 mg of streptomycin/mL), 50 % glucose and 1.4% of HEPES. The cells were kept at room temperature for up to 72 h.

Upon registration of sodium currents, cells were bathed with a combination of two external solutions: NaCl solution (115 mM NaCl, 5 mM KCl, 2 mM CaCl_2_, 1 mM MgCl_2_, 10 mM HEPES, 20 mM TEA-Cl, 0.2 mM CdCl_2_, 0.2 mM NiCl_2_ and 5 mM glucose, pH 7.4) and Choline chloride solution (115 mM ChoCl, 5 mM KCl, 2 mM CaCl_2_, 1 mM MgCl_2_, 10 mM HEPES, 20 mM TEA-Cl, 0.2 mM CdCl_2_, 0.2 mM NiCl_2_ and 5 mM glucose, pH 7.4). The solution for filling the micropipettes was 100 mM CsF, 20 mM NaCl, 10 mM HEPES, 11 mM EGTA, 10 mM TEA-Cl and 5 mM MgCl_2_, pH 7.2. Potassium currents were blocked with the presence of Cs^+^ in internal solution and TEA in the external and internal solutions. Ca^2+^ currents were blocked by Cd^2+^ and Ni^2+^ in external solutions.

Two electrophysiological protocols were used, the first, "test 0", the cell was maintained in a "potential holding" of -80 mV, then a pulse of -120mV was applied with a duration of 100 ms, so that all channels for sodium were close, and just after a test pulse of -20 mV for 100 ms. The second protocol, a -100 mV prepulse was applied with a duration of 100 ms and starting from the potential of -120 mV in increments of 10 mV (up to +30 mV) per 100 ms, to obtain the current/voltage ratio.

*DUM cells*

The DUM cells were isolated from cockroaches *Periplaneta americana*. Ten individuals were euthanized by freezing and the last ganglia from the abdominal nerve cord removed for dissection.

The ganglia were placed in saline solution (200 mM NaCl; 3.1 mM KCl; 5 mM CaCl_2_; 4 mM MgCl_2_; 10 mM HEPES; 50 mM sucrose; pH 7.4) containing 3 mg of collagenase (type 1A from *Clostridium histolyticum*)*,* for 45 min at 37ºC. 

The solution was centrifuged for 25 s at 5000 rpm and the supernatant was discarded. The enzyme was washed 3 times with 3 mL SSAS medium (saline, 2% fetal bovine serum and 1% antibiotic - 5000 units of penicillin and 5 mg of streptomycin/mL). Finally, the cells underwent mechanical grinding, plated and fed, after 2h, with the same medium. The cells were kept at room temperature for up to 24 h.

The cells were bathed with the external solution 100 mM NaCl, 100 mM TEA-Cl, 3.1 mM KCl, 0.05 mM CdCl_2_, 2 mM CaCl_2_, 7 mM MgCl_2_, 4 mM aminopyridine and 20 mM HEPES, pH 7.3. The solution for filling the micropipettes was 15 mM NaCl, 80 mM CsCl, 5 mM EGTA, 10 mM HEPES, 2 mM ATP-Mg_2_, 1 mM MgCl_2_, pH 7.4.

The two electrophysiological protocols used for the DUM cell were: In the first, "test 0", the cell was maintained at a potential holding potential of -90 mV, then a pulse of -120 mV with a duration of 100 ms was applied, so that the sodium channels close, and just after a test pulse of 0 mV for 50 ms. In the second, to obtain a current/voltage ratio, a -100 mV prepulse was applied with a duration of 100 ms and starting from the potential of -120 mV in increments of 10 mV (up to +30 mV) per 100 ms.

## Results

The single sucrose gap was performed with the crude venom (0.754 mg/mL), effects are evidenced by the delay in repolarization and the reduction in the amplitude of the compound action potential, when compared with the control (n = 1) (Figure 1B). Purification of the crude venom by RP-HPLC (C18 analytical column Phenomenex Synergi Hydro 250 x 4.60 mm, detection at 216 nm) presented 67 fractions, with 7 predominant fractions (Figure 1C).

In order to find the neuroactive peptides from the *T. (Archaeotityus) mattogrossensis* venom, the 7 most abundant chromatographic fractions highlighted in Figure1C were tested, individually in single sucrose gap (n = 1), as well as the molecular masses present in these fractions were elucidated by MALDI-TOF mass spectrometry.

**Figure 2.**
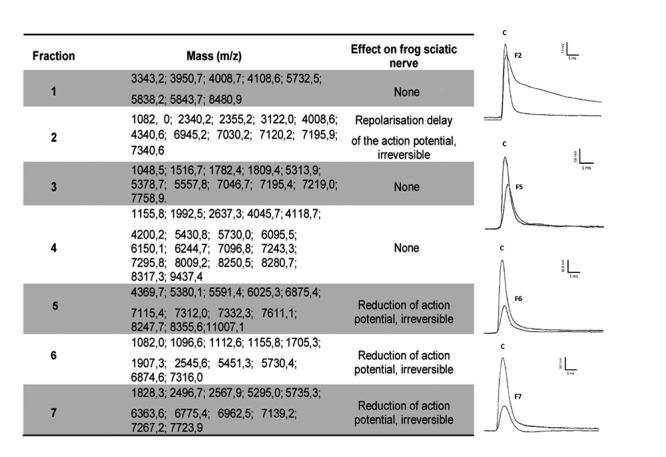
On the left: the summary of the seven most abundant fractions in *Tityus (Archaeotityus) mattogrossensis* venom, masses and the effect on frog sciatic nerve. On the right: electrophysiological analysis (n = 1) of F2, F5, F6 and F7 (0.4 μg/mL of total protein, each) in sucrose gap in frog sciatic nerve, after 40 min of fraction application. F5 (Tm1) shows a 40% reduction in the amplitude of action potential and F6 (Tm2) 62.5% reduction in the amplitude of action potential. C: control test.

F2 caused a delay in repolarization of the action potential, and fractions 5-7 caused a reduction in amplitude of the action potential (Figure 2). 

F5 and F6 (0.4 μg/mL of total protein) reduced 40% and 62.5% of the amplitude of action potential in a frog nerve in single sucrose gap (Figure 2), respectively, which is characteristic of NaScTx. F5 and F6 were chosen for further characterization due to abundance in venom, and renamed as Tm1 and Tm2 respectively.

**Figure 3.**
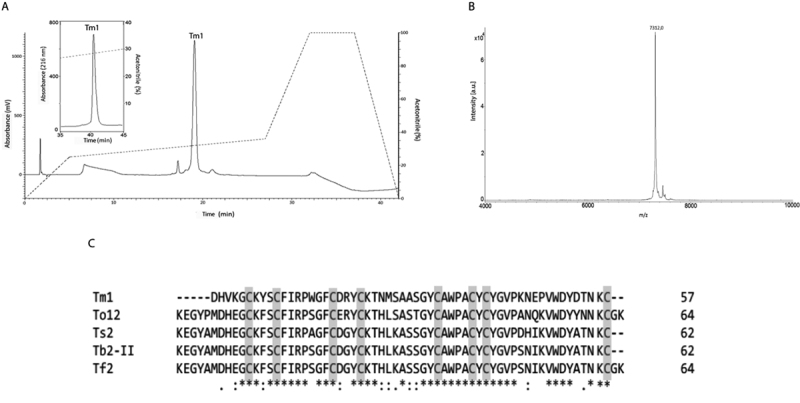
(A) Second step of purification of Tm1 in RP-HPLC analytical column Luna Phenomenex (4.60 x 150 mm, 3 μm). (B) Mass spectrometry MALDI-TOF in positive linear mode, showing the relative degree of purity and the average mass of the peptide. (C) Clustal Omega Tm1 sequence alignment with high similarity scorpion toxins sequences found in BLAST analysis. Cysteine residues are highlighted in gray.

The Tm1 after further purification steps (Figure 3A) presented a single component with an average mass of 7312.0 Da in MALDI-TOF in linear mode (Figure 3B). The *de novo* sequencing by ISD revealed only 8 amino acid residues (Additional file 1). The fragmentation analysis (MALDI-TOF MS/MS) of the tryptic digested peptides by using LIFT mode allowed to infer a partial sequence of 57 amino acid residues (Additional file 2).

The overlapping of the tryptic fragments and the small sequence obtained by ISD resulted in the following partial sequence for Tm1: DHVK/QGCK/QYSCFI/LRPWGFCDRYCK/QTNMSAASGYCAWPACYCYGVPKNEPVWDYDTNKC.The calculation of cysteine residues performed after the reduction and alkylation of the peptide, showed the presence of 8 S-carboxyamidomethylated cysteine residues. With the methodologies used it was not possible to elucidate the N-terminal sequence of Tm1.

Tm1 sequence were submitted to NCBI protein database obtaining, 75% similarity with *Tityus bahiensis* Tb2-II toxin (P60276), 75% with *Tityus fasciolatus* Tf2 (C0HJM9), 74% with *Tityus obscurus* To12 (H1ZZI1), and 74% *Tityus serrulatus* Ts2 toxin (P68410). The mature toxins were alignment done using the Clustal Omega (Figure 3C).

The sequence and alignment suggest that Tm1 is a NaScTx, however, in order to know the real activity of this neurotoxic peptide, electrophysiological tests were performed with 4 types of neuronal cells in a patch clamp: immortalized HEK293 Nav 1.3 and HEK293 Nav 1.6, DRG and DUM.

**Figure 4.**
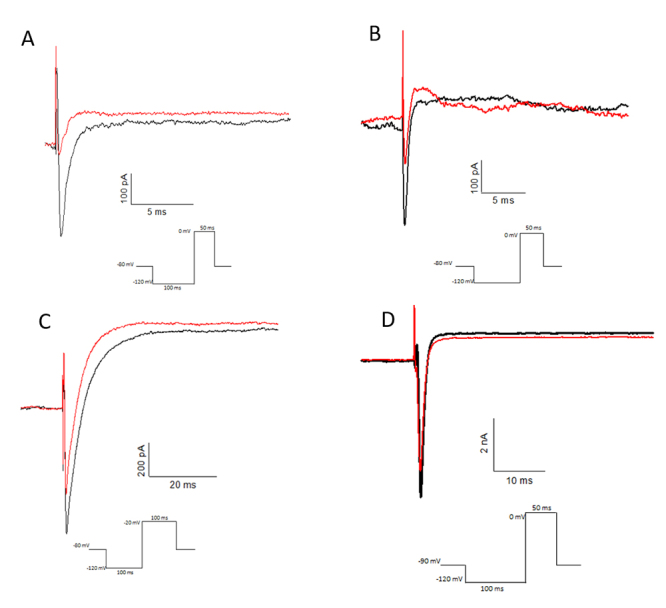
Tm1 (1 µM) toxin activity (red) compared to the control (black) in whole-cell patch clamp with: **(A)** HEK293 Nav 1.3, showing a 71.5% (n = 1) reduction in current; **(B)** HEK293 Nav 1.6 showing a 63.3 ± 7% (n = 3) reduction in current; **(C)** DRG showing a 13.2 ± 7% (n = 2) reduction in current; **(D)** DUM showing a 35 ± 4.1% (n = 5) reduction in current.

The electrophysiological results obtained with Tm1 (1 µM) showed a sodium current reduction activity, in test protocol "test 0", of 71.5% (n = 1) in Nav 1.3 channels (Figure 4A) and 63.3 ± 7% (n = 3) reduction in Nav 1.6 (Figure 4B). Tm1 (1 µM) also inhibited the sodium currents in DRG cells in 13.2% (n = 2) (Figure 4C). And in DUM cells, Tm1(1 µM) showed a considerable effect of 35 ± 4.1% (n = 5) of sodium current reduction (Figure 4D). In all assays the action of Tm1 was irreversible. In measuring voltage vs current in HEK298 Nav 1.6 cells, the shift to more negative potentials was not observed, which indicates that Tm1 is not a β-NaScTx (Additional file 3).

**Figure 5.**
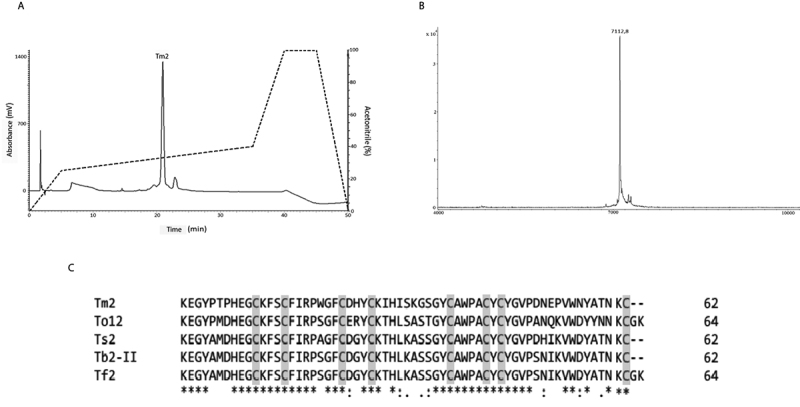
(A) Second step of purification of Tm2 in RP-HPLC analytical column Luna Phenomenex Luna C18 (4.60 x 150 mm, 3 μm) detection at 216 nm, flow of 1 mL/min, optimized gradient of acetonitrile (dotted line). (B) Mass spectrometry (MALDI-TOF/MS) of Tm2, showing the degree of purity and the average mass. (C) Alignment of the Tm2 sequence with the high identity sequences of other scorpion toxins present in the database. Cysteine residues are highlighted in gray.

The second purification step of Tm2 is presented in Figure 5A. The degree of purity and the average mass of 7112.8 Da was obtained by MALDI-TOF/MS in the positive linear mode (Figure 5B). And the monoisotopic mass of 7108.0Da was defined by MicrOTofQII, and calculated using the Bruker Compass software.

Tm2 peptide was also sequenced in two stages: 37 amino acid residues were obtained by ISD (Additional file 4); and four tryptic fragments by LIFT mode (Additional file 5).

The calculation of cysteines performed after the reduction and alkylation of the peptide, showed the presence of 8 S-carboxyamidomethylated cysteine residues. The partial sequence obtained for Tm2 was K/QEGYPTPHEGCKFSCFI/LRPWGFCDHYCKI/LHI/LSK/QGSGYCAWPACYCYGVPDNEPVWNYATNKC.

Tm2 sequence were submitted to BLAST database obtaining 75% similarity with *Tityus bahiensis* Tb2-II toxin (P60276), *Tityus fasciolatus* Tf2 (C0HJM9), *Tityus serrulatus* Ts2 (P68410); and 71% similarity with *Tityus obscurus* To12 (H1ZZI1) (Figure 5C).

As Tm2 sequence has a high identity (>75%) with NaScTx, this peptide was also submitted to patch clamp tests were performed with HEK298 Nav 1.3 and Nav 1.6, and DRG cell types. The DUM test was not performed.

**Figure 6.**
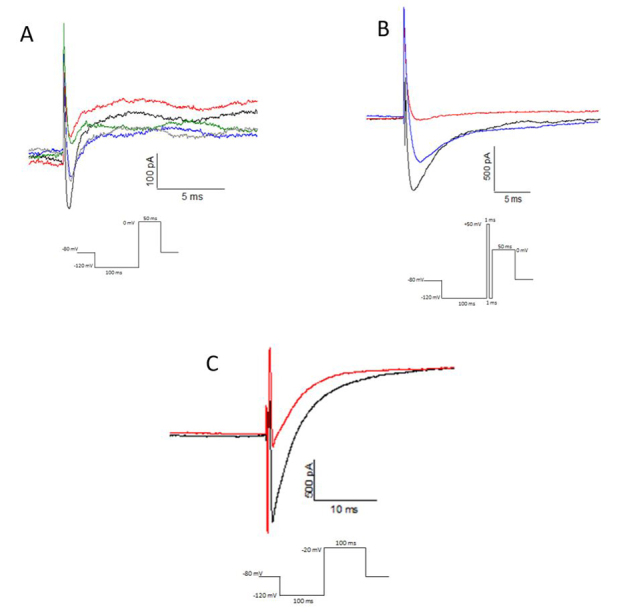
Tm2 (1 µM) toxin activity (red) compared to the control (black) in whole-cell patch clamp with: **(A)** HEK293 Nav 1.3, showing 35.81 ± 10.2% (n = 5) reduction in current partial washing (blue), Tm2 (1 µM) reperfused (green) and washed (gray); **(B)** HEK293 Nav 1.3 cell, in depolarizing prepulse +50 mV for 1 ms protocol, showing 95.15 ± 3.65% (n = 2) reduction in current, partial washing (blue); **(C)** DRG showing 25% (n = 1), reduction in current.

Tm2 toxin (1μM) showed partially reversible activity, reducing 35.81 ± 10.2% (n = 5) of the sodium current in HEK298 Nav 1.3 cells (Figure 6A). The activity was intensified by applied the depolarizing prepulse of +50 mV, which became an inhibition of 95.15 ± 3.65% (n = 2) (Figure 6B). Tm2 (1μM) showed the activity of reducing the sodium current by 25% activity in DRG cells which (Figure 6C). Tm2 showed no activity in HEK298 Nav 1.6 cells (data not shown).

In measuring voltage vs current in HEK298 Nav 1.3 cells, there were no shift to more negative potentials (Additional file 6), and the intensification of the action of Tm2 by the pre-pulse indicates Tm2 is a α-NaScTx.

**Figure 7.**
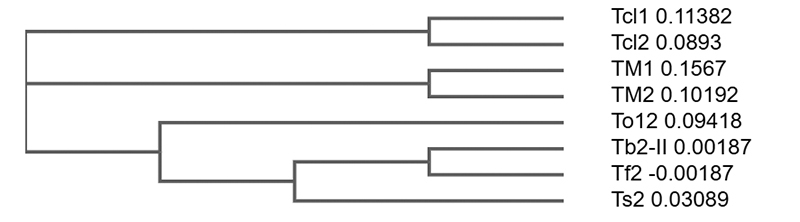
Phylogenetic tree obtained from Tm1 and Tm2 from *Tityus (Archaeotityus) mattogrossensis* isolated in this work, and Tcl1 *Tityus (Archaeotityus) clathratus* (J9PJ66) and Tcl2 *Tityus (Archaeotityus) clathratus* (J9PIJ6), *Tityus bahiensis* Tb2-II toxin (P60276), *Tityus fasciolatus* Tf2 (C0HJM9), *Tityus serrulatus* Ts2 (P68410); *Tityus obscurus* To12 (H1ZZI1). CLUSTAL Omega multiple sequence alignments is presented in Additional file 7.

A phylogenetic analysis was performed to investigate the phylogenetic relationships of Tm1 and Tm2 from *Tityus (Archaeotityus) mattogrossensis* toxins with *T. (Archaeotityus) clathratus* and other *Tityus* NaScTxs. Figure 7 shows a consensus tree obtained after Clustal Omega alignment. *Tityus (Archaeotityus) mattogrossensis* clade is placed between *T. (Archaeotityus) clathratus* and *Tityus* subgenus.

## Discussion

*Tityus (Archaeotityus) mattogrossensis* crude venom showed to be a complex mixture with 67 fractions separated in RP-HPLC, also the crude venom promoted a delay in repolarisation and the reduction in the amplitude of the compound action potential.

The peptide sequences identified in the present work, Tm1 and Tm2, showed high identity with other NaScTxs described from scorpions of the subgenus *Tityus.* Tm1 toxin showed 75% similarity with Tb2-II toxin and Tf2; and 74% similarity with Ts2 and To12, respectively. 

Tm2 peptide sequence showed 75% similarity with Tb2-II, Tf2 and Ts2 and also presented 71% similarity with To12.

Pimenta and collaborators (2001) [15] described the *Tityus bahiensis* Tb2-II (P60276) activity in mammals and in insects. *Tityus serrulatus* Ts2 (P68410) is a α-NaScTx and inhibits the inactivation of the activated channels, thereby blocking neuronal transmission [16]. It has been shown the specificity and selectivity of Ts2 for some mammalian sodium channels inhibiting rapid inactivation of Na_V_1.2, Na_V_1.3, Na_V_1.5, Na_V_1.6 and Na_V_1.7, but does not affect Na_V_1.4, Na_V_1.8 or the insect sodium channel (*Drosophila melanogaster*sodium channel **-** DmNa_V_1) [16].

*Tityus fasciolatus* Tf2 (C0HJM9) also have a similar sequence to Tb2-II (P60276), and has been shown an activity in human hNav1.3 [17] shifting the activation voltage to much more negative values, effectively opening the channel at resting membrane potentials.

*Tityus obscurus* To12 (H1ZZI1) is a β-NaScTx. It shifts the voltage of activation toward more negative potentials thereby affecting sodium channel activation and promoting spontaneous and repetitive firing [18].

The amino acid sequences of Tm1 and Tm2 toxins are similar (Additional file 7) to each other and with other NaScTx [18,19] which present activity in mammals and insects sodium channels, although the NaScTx primary sequence does not categorize them as α-NaScTx or β-NaScTx, this difference is only observed experimentally where the β-NaScTx cause a more negative potential shift.

Only Tm1 showed activity in Nav 1.6 channels, reduction of 63.3 ± 7% (n = 3) of the current with 1 µM of toxin. This special difference may be attributed to some residues in C- and N-terminal portion of Tm1. Unfortunately, it was not possible to sequence the Tm1 N-terminal Tm1, which could bring more information about its structure/activity. However, we bring to light the difference after the alignment of the cysteines, it is observed the presence of a valine in position 8, which occurs only in Tm1 and not observed in Tm2 or in the other toxins aligned.

In DUM cell channels Tm1 also reduced 35 ± 4.1% of the current with 1 µM of toxin. DUM neurons are important for locomotion, neuromodulation and secreting octopamine that changes the basic tension used in different metabolic substrates of insects [20]. There are only two studies on the activity of scorpionic toxins in DUM cells, α-NaScTx described by *Buthus martensi* (BmK M1) and other with *Buthus occitanus tunetunus* (BotIT2) [21,22]. Currently, most assays for insecticidal toxins are *in vivo* which does not indicate their specific pharmacological action or their molecular target, demonstrating the need for further studies using the "patch clamp" technique with insect neuronal cells (DUM).

Tm1 and Tm2, showed sodium current reduction activity in HEK293 Nav 1.3 cells of 71.5% (n = 1) and 35.81 ± 10.2% (n = 5), Tm2 was also tested in HEK293 Nav 1.3 cells with a +50 mV depolarizing pulse protocol, the activity of the toxin increased from 35.81 ± 10.2% (n = 5) to 95.15% (n = 2). This activity of sudden decrease in current with the presence of a previous depolarizing pulse is uncommon in β-NaScTx. Previous depolarization protocols increase the current in more negative potentials in β-NaScTx assays [23]. This is due to the induction of a conformational change in channel II domain voltage sensor, generated by the pre-pulse, thereby promoting an increase in the binding force of the toxin. In this case, the toxin binding prevents the voltage sensor return to the resting state and reduces the energy needed to open the channel [24]. This difference in amino acids in the α-helix region, which forms the loop of the molecule, which is responsible for the interaction with the sodium channels, may be the reason why the Tm2 toxin showed an unusual activity when pre-pulsating cells HEK293 Nav 1.3, more detailed electrophysiological tests should be carried out to resolve this question. Although Tm1 and Tm2 also promote no shift to more negative potentials, that strongly suggesting that both are α-NaScTx.

The toxins’ phylogenetic relationships showed a cluster of *Tityus (Archaeotityus) mattogrossensis* (Tm1 and Tm2) between subgenus *Tityus* (Tb2-II, Tf2, Ts2 and To12) and *Tityus (Archaeotityus) clathratus* (Tcl1and Tcl2). Although a molecular analysis of more *Archaeotityus* species is needed to evaluate whether they to determine their phylogenetic relation with species in the subgenus *Tityus*.

## Conclusion

Although *Tityus (Archaeotityus) mattogrossensis* is considered as an ancient group in *Tityus* genus, the primary structure of the two isolated toxins (Tm1 and Tm2) is more related to *Tityus* subgenus. Tm1 and Tm2 are NaScTx, in patch clamp electrophysiological tests promoting reduction on HEK293 Nav 1.3, DUM and DRG cells. More specifically, only Tm1 had activity on HEK293 Nav 1.6. Tm1 and Tm2 also promote no shift to more negative potentials, strongly suggesting that both are α-NaScTx.

Studies with scorpion toxins are closely related to their sting toxicity and consequent deleterious effects on humans. However, scorpion species not considered to be of medical importance can provide important information about the diversity and evolution of toxin structures.

## Supplementary material

The following online material is available for this article:

Additional file 1. Sequencing of F5 by MALDI-TOF/MS with ISD fragmentation showed a peptide with partial sequence of eight amino acid residues.

Additional file 2. Sequences of the F5 tryptic digestions. The fragmentation was done in lift mode by MALDITOF/MS.

Additional file 3. (A) Current (nA) and voltage (mV) ratio graph of the Tm1 (1 µM) activity (red); control (black). There was no shift to more negative potentials in relation to the control. (B) Protocol performed to obtain the current X voltage (IxV) register. (C) Sodium current recording from the same cell in which the currents of the IxV graph were recorded, in black the control sodium current and in red the current under the action of Tm1 (1 µM).

Additional file 4. Sequencing of F6 by MALDI-TOF with ISD fragmentation showed a peptide with partial sequence of 37 amino acid residues.

Additional file 5. Sequences of the F6 tryptic digestions. The fragmentation was done in lift mode by MALDI-TOF/MS.

Additional file 6. (A) Current (nA) and voltage (mV) ratio graph of Tm2 (1 µM) activity (red) in HEL293 Nav 1.6 cell; control (black). There was no shift to more negative potentials in relation to the control. (B) Protocol performed to obtain the current X voltage (IxV) register. (C) Sodium current recording from the same cell in which the currents of the IxV graph were recorded, in black the control sodium current and in red the current under the action of Tm2 (1 µM).

Additional file 7. Clustal Omega alignment of Tm1 and Tm2 isolated in this work and *Tityus bahiensis* Tb2-II toxin (P60276), *Tityus fasciolatus* Tf2 (C0HJM9), *Tityus serrulatus* Ts2 (P68410); *Tityus obscurus* To12 (H1ZZI1), Tcl1 *Tityus clathratus* (J9PJ66) and Tcl2 *Tityus clathratus* (J9PIJ6). (*****) Indicates identical residues, (**:**) indicates strong conservation, (**.**) indicates weak conservation sites.
