# Supplementary material for: Characterization of the first two toxins isolated from the venom of the ancient scorpion Tityus (Archaeotityus) mattogrossensis (Borelli, 1901)
Source: J Venom Anim Toxins Incl Trop Dis. 2021 Dec 13;27:e20210035. doi: 10.1590/1678-9199-JVATITD-2021-0035 (PMC8670738; doi:10.1590/1678-9199-JVATITD-2021-0035)

*J Venom Anim Toxins incl Trop Dis*

https://doi.org/10.1590/1678-9199-JVATITD-2021-0035

# Supplementary Material to "Characterization of the first two toxins isolated from the venom of the ancient scorpion *Tityus (Archaeotityus) mattogrossensis* (Borelli, 1901)

**Additional file 1.** Sequencing of F5 by MALDI-TOF/MS with ISD fragmentation showed a peptide with partial sequence of eight amino acid residues.

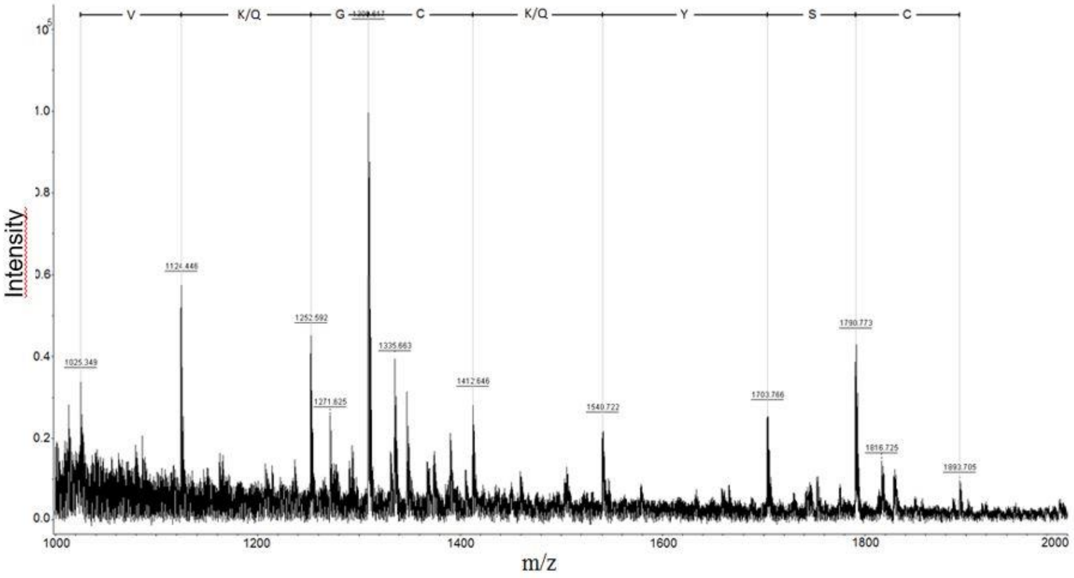

Supplement: Additional file 1. [file 1678-9199-jvatitd-27-e20210035-s1.pdf]
