# Supplementary material for: Characterization of the first two toxins isolated from the venom of the ancient scorpion Tityus (Archaeotityus) mattogrossensis (Borelli, 1901)
Source: J Venom Anim Toxins Incl Trop Dis. 2021 Dec 13;27:e20210035. doi: 10.1590/1678-9199-JVATITD-2021-0035 (PMC8670738; doi:10.1590/1678-9199-JVATITD-2021-0035)
Supplement: Additional file 2. [file 1678-9199-jvatitd-27-e20210035-s2.pdf]

J Venom Anim Toxins incl Trop Dis

https://doi.org/10.1590/1678-9199-JVATITD-2021-0035

Supplementary Material to "Characterization of the first two toxins isolated from the venom of the ancient scorpion *Tityus (Archaeotityus) mattogrossensis* (Borelli, 1901)

Additional file 2. Sequences of the F5 tryptic digestions. The fragmentation was done in lift mode by MALDITOF/MS.

Fragment masses (m/z)	Fragment sequences
**654.19**	GFCDR
**845.33**	YSCFI/LR
**937.33**	PWGFCDR
**1046.38**	CYCYGVPK
**1128.46**	YSCFI/LRPW
**1380.56**	NEPVWDYDTNK
**1763.74**	YSCFI/LRPWGFCDR
**2513.91**	TNMSAASGYCAWPACYCYGVPK
**4024.64**	DHVK/QGCK/QYSCFI/LRPWGFCDRYCK/QTNMSAASGY
