# Supplementary material for: Characterization of the first two toxins isolated from the venom of the ancient scorpion Tityus (Archaeotityus) mattogrossensis (Borelli, 1901)
Source: J Venom Anim Toxins Incl Trop Dis. 2021 Dec 13;27:e20210035. doi: 10.1590/1678-9199-JVATITD-2021-0035 (PMC8670738; doi:10.1590/1678-9199-JVATITD-2021-0035)

J Venom Anim Toxins incl Trop Dis

https://doi.org/10.1590/1678-9199-JVATITD-2021-0035

Supplementary Material to "Characterization of the first two toxins isolated from the venom of the ancient scorpion *Tityus (Archaeotityus) mattogrossensis* (Borelli, 1901)

Additional file 3. (A) Current (nA) and voltage (mV) ratio graph of the Tm1 (1 µM) activity (red); control (black). There was no shift to more negative potentials in relation to the control. **(B)** Protocol performed to obtain the current X voltage (IxV) register. **(C)** Sodium current recording from the same cell in which the currents of the IxV graph were recorded, in black the control sodium current and in red the current under the action of Tm1 (1 µM).

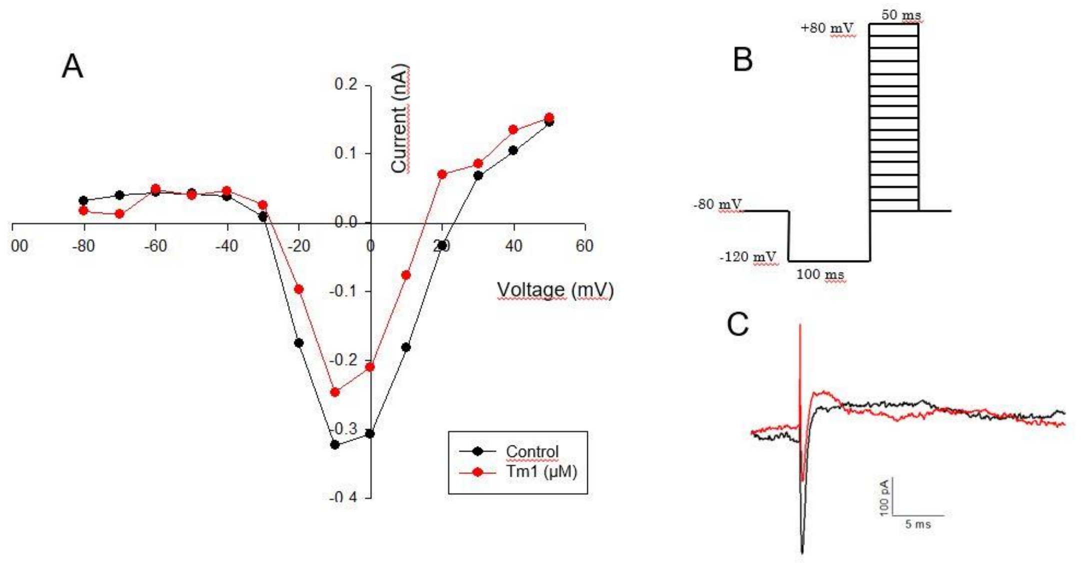

Supplement: Additional file 3. [file 1678-9199-jvatitd-27-e20210035-s3.pdf]
