# Supplementary material for: Characterization of the first two toxins isolated from the venom of the ancient scorpion Tityus (Archaeotityus) mattogrossensis (Borelli, 1901)
Source: J Venom Anim Toxins Incl Trop Dis. 2021 Dec 13;27:e20210035. doi: 10.1590/1678-9199-JVATITD-2021-0035 (PMC8670738; doi:10.1590/1678-9199-JVATITD-2021-0035)
Supplement: Additional file 5. [file 1678-9199-jvatitd-27-e20210035-s5.pdf]

J Venom Anim Toxins incl Trop Dis

https://doi.org/10.1590/1678-9199-JVATITD-2021-0035

Supplementary Material to "Characterization of the first two toxins isolated from the venom of the ancient scorpion *Tityus (Archaeotityus) mattogrossensis* (Borelli, 1901)

Additional file 5. Sequences of the F6 tryptic digestions. The fragmentation was done in lift mode by MALDI-TOF/MS.

**Fragment masses (m/z)**	**Fragment sequences**
**1402,57**	K/QEGYPTPHEGCK
**1489,67**	I/LHI/LSK/QSGYCAWPA
**1703,75**	GVPDNEPVWNYATNK
**2179,86**	FSCFI/LRPWGFCDHYCK
