# Supplementary material for: Characterization of the first two toxins isolated from the venom of the ancient scorpion Tityus (Archaeotityus) mattogrossensis (Borelli, 1901)
Source: J Venom Anim Toxins Incl Trop Dis. 2021 Dec 13;27:e20210035. doi: 10.1590/1678-9199-JVATITD-2021-0035 (PMC8670738; doi:10.1590/1678-9199-JVATITD-2021-0035)
Supplement: Additional file 7. [file 1678-9199-jvatitd-27-e20210035-s7.pdf]

J Venom Anim Toxins incl Trop Dis

https://doi.org/10.1590/1678-9199-JVATITD-2021-0035

Supplementary Material to "Characterization of the first two toxins isolated from the venom of the ancient scorpion *Tityus (Archaeotityus) mattogrossensis* (Borelli, 1901)

Additional file 7. Clustal Omega alignment of Tm1 and Tm2 isolated in this work and *Tityus bahiensis* Tb2-II toxin (P60276), *Tityus fasciolatus* Tf2 (C0HJM9), *Tityus serrulatus* Ts2 (P68410); *Tityus obscurus* To12 (H1ZZI1), Tcl1 *Tityus clathratus* (J9PJ66) and Tcl2 *Tityus clathratus* (J9PIJ6). (*****) Indicates identical residues, (**:**) indicates strong conservation, (**.**) indicates weak conservation sites.

```
CLUSTAL O(1.2.4) multiple sequence alignment


Tcl1    KEGYLMDHEGCKLSCFIRPAGYCGRECSIK-KGKNGYCRWPACYCYDLPGWAKVWDRATNRCGKK 64
Tcl2    KEGYLMDREGCKLSCFIRPSGYCGRECEIK-KGSSGYCRWLACYCYGLPDRVKVWSYATNTCGKK 64
TM1     ----MDHVKGCKYSCFIRPWGFCDRYCKTNMSAASGYCAWPACYCYGVPKNEPVWDYDTNKC--- 58
TM2     KEGYPTPHEGCKFSCFIRPWGFCDHYCKIHISKGSGYCAWPACYCYGVPDNEPVWNYATNKC--- 62
To12    KEGYPMDHEGCKFSCFIRPSGFCERYCKTHLSASTGYCAWPACYCYGVPANQKVWDYYNNKCGK- 64
Tb2-II  KEGYAMDHEGCKFSCFIRPSGFCDGYCKTHLKASSGYCAWPACYCYGVPSNIKVWDYATNKC--- 62
Tf2     KEGYAMDHEGCKFSCFIRPSGFCDGYCKTHLKASSGYCAWPACYCYGVPSNIKVWDYATNKCGK- 64
Ts2     KEGYAMDHEGCKFSCFIRPAGFCDGYCKTHLKASSGYCAWPACYCYGVPDHIKVWDYATNKC--- 62
                 :*** ****** *:*   *. : .  .*** * *****.:*    **.  .**
```
